# Long-term safety of mepolizumab for up to ∼10 years in patients with severe asthma: open-label extension study

**DOI:** 10.1080/07853890.2024.2417184

**Published:** 2024-10-28

**Authors:** Ian Pavord, Robert Chan, Nicola Brown, Peter Howarth, Martyn Gilson, Robert G. Price, Jorge Maspero

**Affiliations:** a Respiratory Medicine Unit and Oxford Respiratory National Institute for Health Research Biomedical Research Centre, Nuffield Department of Medicine, University of Oxford, Oxford, UK; b Clinical Sciences, Respiratory, GSK, London, UK; c Global Medical, Specialty Medicine TA, GSK, London, UK; d Respiratory Research and Development, GSK, Stevenage, Hertfordshire, UK; e Biostatistics, GSK, Stevenage, Hertfordshire, UK; f Clinical Investigation, Allergy and Respiratory Research Unit, Fundacion CIDEA, Buenos Aires, Argentina

**Keywords:** Long-term access program, mepolizumab, open-label extension, safety, severe asthma with an eosinophilic phenotype

## Abstract

**Objectives:**

Long-term safety monitoring of mepolizumab is necessary to support real-world use for the treatment of severe asthma. This Long-Term Access Program assessed the safety and benefit:risk of mepolizumab in pediatric, adolescent, and adult patients with severe asthma.

**Materials and methods:**

This was a multicenter, Phase IIIb safety, open-label extension study of multiple prior studies assessing mepolizumab in addition to standard of care (Aug 2015 − Aug 2022). Adults/adolescents (≥12 years of age) received mepolizumab 100 mg subcutaneously (SC) every 4 weeks until mepolizumab was commercialized. Pediatric patients (6–11 years of age) received mepolizumab 40 mg or 100 mg SC (bodyweight <40 or ≥40 kg, respectively) every 4 weeks. Safety was assessed every 4 weeks and benefit:risk every 12 weeks.

**Results:**

Of the 514 patients enrolled, 57% were female and the mean age was 51.1 (standard deviation: 14.9) years; 24 (5%) patients were 6–17 years of age. Total cumulative mepolizumab exposure across all mepolizumab studies included in this analysis was 1500.59 patient-years; median exposure was 2.03 (range, 0.08 to 9.97) years. Overall, 37 (7%) patients experienced on-treatment serious adverse events (SAEs): 34/502 (7%) in the 100 mg SC group and 3/7 (43%) in the 40 mg SC pediatric group. Two patients experienced SAEs considered to be treatment-related by the investigator. Infections were the most common SAEs of special interest (9 [2%] patients). Physician-assessed benefit:risk of mepolizumab supported continued treatment over the study period.

**Conclusions:**

This long-term safety analysis of mepolizumab was consistent with previous reports, with no emerging safety concerns; most patients had a favorable benefit:risk up to ∼10 years.

**Clinical trial identifier:**

NCT00244686 (GSK ID 201956)

## Introduction

Asthma is a common, heterogeneous condition marked by airway hyperresponsiveness and wheezing that is estimated to affect around 358 million people worldwide [1, 2]. While data are limited, it is generally estimated that severe asthma affects 5–10% of patients with asthma [3] and is associated with increased morbidity and mortality compared with non-severe asthma [3–5]. It has been suggested based on sputum estimates that around 50% of patients with severe asthma have airway eosinophilia [6–9]. However, airway eosinophilia estimates from sputum are likely an underestimate, as degranulated eosinophils are not included in the differential count [9], and, repeated measures in blood identify more patients as having an eosinophilic phenotype than established estimates [10]. In a recent study by Heaney et al., the eosinophilic phenotype in patients with severe asthma was suggested to be more prevalent than previous estimates, with over 80% of patients identified as having eosinophilic airway inflammation [11]. The functional implications of eosinophilic inflammation are highlighted by its association with increased risk of severe exacerbations [6–8, 12]. Consequently, given that eosinophils play an important part in airway inflammation in asthma and that interleukin-5 (IL-5) is the primary cytokine involved in eosinophil differentiation, activation and survival [13–16], therapies that target IL-5 provide a precision-medicine approach for severe asthma with an eosinophilic phenotype [17, 18]. IL-5 is thus fundamental to severe asthma, and this understanding is recognized in the guideline recommendations for biologic therapies that target IL-5 in the management of this disease [19–21].

Mepolizumab is a humanized anti-IL-5 monoclonal antibody and is approved for use in severe asthma with an eosinophilic phenotype, eosinophilic granulomatosis with polyangiitis, hypereosinophilic syndrome, and chronic rhinosinusitis with nasal polyps for various age groups in many countries worldwide, including the European Union and the USA [22, 23]. Treatment of severe asthma with mepolizumab reduces airway and sputum eosinophils [24], and with the licensed 100 mg subcutaneous (SC) dose [22, 23], reduces severe disease exacerbations by up to 53% compared with placebo in randomized controlled trials and 78% compared with the year prior to therapy in real-world use [25, 26]. Additionally, mepolizumab treatment results in better asthma control, reduced oral corticosteroid use, and improved lung function and quality of life [17]. Furthermore, mepolizumab has demonstrated a favorable safety profile in randomized placebo-controlled trials [24, 26–28]. While mepolizumab is approved for pediatric patients 6 to <18 years of age with severe asthma in several countries, there remains a paucity of data within this age group [29–32].

This study aimed to provide long-term access to mepolizumab for those patients with severe asthma who had previously been in a mepolizumab trial, while also expanding on long-term safety data collected in previous long-term studies; the trials that have fed into this study are summarized in Supplementary Table 1. The study also assessed the benefit:risk of mepolizumab in individual patients with severe asthma, including in pediatric and adolescent patients, for up to ∼10 years.

## Methods

### Study design

This was a multicenter, open-label extension (OLE), Phase IIIb long-term safety study in patients who had previously participated in a clinical trial of mepolizumab in severe asthma (GSK ID: 201956, NCT00244686). The trials patients were previously enrolled in were DREAM (NCT01000506), COLUMBA (NCT01691859), COMET (NCT02555371), SIRIUS (NCT01691508), COSMOS (NCT01842607), COSMEX (NCT02135692), MENSA (NCT01691521), MUSCA (NCT02281318), a pediatric study (NCT02377427), and OSMO (NCT02654145), details of which are in Supplementary Table 1. The aim of this Long-Term Access Program (LAP) was to provide open-label mepolizumab on an individual basis to eligible patients with asthma who had previously participated in a GSK-sponsored clinical trial for mepolizumab within the last 6 months; those >6 months since their last visit were considered on an individual basis. Mepolizumab was provided from August 2015 until it was commercially licensed for the treatment of severe asthma for the age-specific population in the relevant country, or until the program closed in August 2022 (protocol predefined closure date). This study was performed in accordance with International Council for Harmonisation, Good Clinical Practice, the Declaration of Helsinki and applicable country-specific regulatory requirements. Institutional Review Board/Institutional Ethics Committee approvals were obtained prior to the study start. All patients provided written informed consent prior to participation in the study. For pediatric patients, parents or guardians provided consent.

### Treatments

Mepolizumab was provided in addition to standard-of-care treatment. Adults/adolescents (≥12 years of age) received mepolizumab 100 mg SC every 4 weeks. Pediatric patients (6–11 years of age) received mepolizumab 40 mg SC every 4 weeks or 100 mg SC every 4 weeks if their bodyweight was ≥40 kg. From June 2020, the pediatric dose was modified so that all patients received 40 mg SC every 4 weeks regardless of weight. The 40 mg SC every 4 weeks pediatric dose was chosen because it was predicted to provide similar exposure to the 100 mg SC dose received every 4 weeks in adults and adolescents. The treating physician was required to perform a benefit:risk assessment every 12 weeks, which was recorded within the Electronic Case Record Form and the treating physician made a judgment on whether to permit continued supply of mepolizumab treatment. If at any assessment the risk:benefit was no longer positive, as determined by the treating physician, then the patient discontinued mepolizumab and the study. Patients were required to withdraw from the study following discontinuation of study treatment.

### Patients

Patients were included in the study if they had participated in a clinical trial of mepolizumab in severe asthma (Supplementary Table 1). This included patients who had completed a prior mepolizumab trial or had been required to withdraw due to study closure prior to commercial availability. Females of childbearing potential were required to use suitable contraceptive measures during the consent period, mepolizumab treatment and for 4 months following the last treatment.

Exclusion criteria included current malignancy or history of cancer in remission for <12 months (except for localized dermal basal or squamous cell carcinoma that had been resected for cure); clinically significant medical conditions not associated with asthma that were uncontrolled with standard-of-care therapy, for example, unstable liver disease, uncontrolled cardiovascular (CV) disease, ongoing active infectious disease requiring systemic treatment; pregnancy, planned pregnancy or breastfeeding, and known allergy or intolerance to a monoclonal antibody or biologic therapy. Other exclusion criteria included patients who had experienced permanent withdrawal of study treatment due to a serious or non-serious adverse event (AE) considered related to treatment in a mepolizumab clinical study, who had received treatment with another biological therapy, who had received an investigational drug within the past 30 days or 5 terminal phase half-lives of the drug (whichever was longer), or who were participating in any other interventional clinical study.

### Endpoints and assessments

Demographic characteristics, cardiovascular medical history and risk assessment, review of AEs and serious AEs (SAEs) and the benefit:risk assessment of mepolizumab were recorded at screening, up to 4 weeks before baseline. Every 4 weeks, pregnancy testing was performed and AEs/SAEs (including duration, severity, causality, action taken and outcome) were recorded; every 12 weeks a benefit:risk assessment was performed. The primary endpoint was the frequency of AEs and SAEs, including all AEs of special interest (AESI; e.g. systemic and local injection-site reactions, serious cardiac, vascular and thromboembolic events, and ischemic events; as well as potential opportunistic infections and malignancies).

### Withdrawal/stopping criteria

Patients continued to receive mepolizumab until any of the following criteria applied: withdrawal from the LAP due to the commercial availability of mepolizumab in the patient’s age-specific population; physician decision that the patient was no longer deriving clinical benefit from treatment or the benefit:risk assessment does not support continued treatment; a treatment-limiting AE(s) and pregnancy or intention to become pregnant (although it should be noted that mepolizumab is not contraindicated during pregnancy by the US Food and Drug administration, while the European Medicines Agency suggest it should be used only when the expected benefit to the mother exceeds possible fetal risks) [22, 23].

### Sample size and statistical analysis requesting

There was no pre-specified sample size for this LAP. The population was instead determined by the number of patients who were eligible and wished to receive continued access to mepolizumab treatment. Continuous data were summarized using descriptive statistics. Categorical data were summarized as the number and percentage of patients in each category. The safety population included all patients who receive ≥1 dose of mepolizumab. For an extensive safety analysis, a non-serious AE population was also included. This population was a subset of the safety analysis set and included all patients who received ≥1 dose of mepolizumab and were required per protocol to report non-serious AEs. Patients were from the UK, Czech Republic, Estonia, Greece, or Slovakia, or had previously been enrolled in the pediatric mepolizumab pharmacokinetics and pharmacodynamics study in children 6–11 years of age (study 200363, NCT02377427) [30, 31]; for the remaining participants, only SAE data were captured during the course of the study.

## Results

### Patient population

The study enrolled 514 patients (502 receiving mepolizumab 100 mg SC, 7 receiving the pediatric dose of mepolizumab 40 mg SC, and 5 who transitioned from 40 mg SC to 100 mg SC during the study follow-up due to age [≥12 years of age] or increasing weight [≥40 kg], who were all included in the safety population). Eighty-eight (17%) patients were included in the non-serious AE population, which included 76/502 (15%) of the mepolizumab 100 mg SC group; 7/7 (100%) of the mepolizumab 40 mg SC pediatric dose group and 5/5 (100%) of the 40/100 mg SC group.

**Figure 1.**
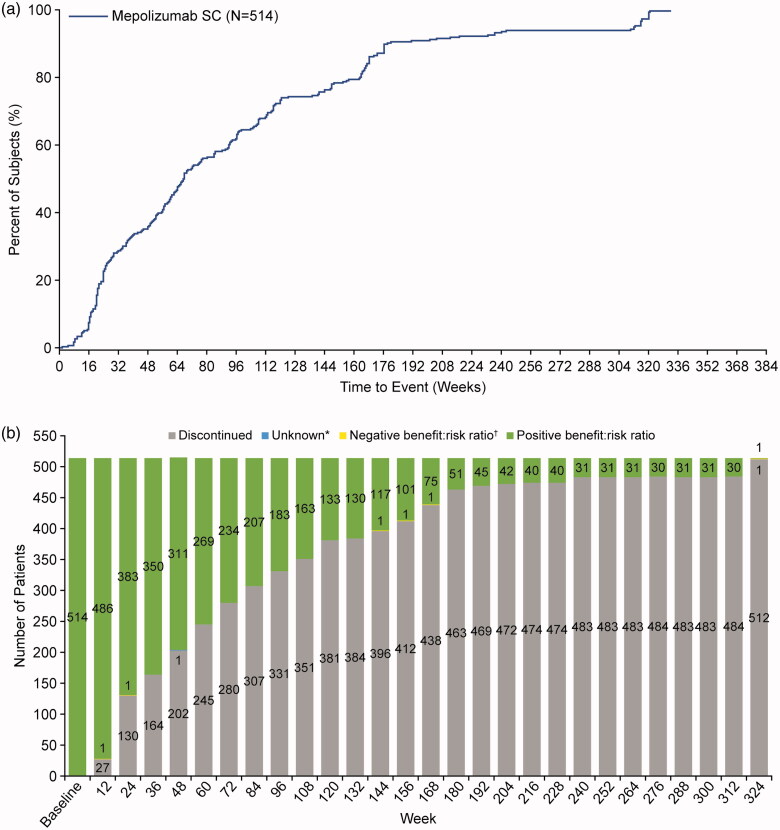
(a) Time to study withdrawal in the open-label extension and (b) physician assessment of benefit:risk ratio. Kaplan–Meier estimates of time to withdrawal from study. Patients are represented from Day 1 to the day of withdrawal from study. *1 patient reported an unknown (missed) benefit:risk assessment at Week 48. ^†^In five patient cases (Weeks 12, 24, 144 and 156 [same individual], 168, and 324), physicians decided the benefit:risk ratio was negative. Where a negative benefit:risk ratio was identified by the treating physician, the patient was subsequently withdrawn from the study. SC: subcutaneous.

Time to study withdrawal from this LAP is presented in Figure 1(a). Overall, 365/514 (71%) of withdrawals from the LAP related to protocol-specified withdrawal criteria being met, the majority of which (360/514, 70%) were as a result of mepolizumab becoming commercially available in the respective age groups, corresponding to study completion. Other reasons for withdrawal prior to study completion included physician decision (68/514 [13%] patients), patient decision (51/514 [10%] patients), lack of efficacy (13/514 [3%] patients) as decided by the treating physician, loss to follow-up (6/514 [1%] patients), study terminated by sponsor (6/514 [1%] patients), and AEs (5/514 [<1%] patients). Sixty-three of all 91 patients from the Ukraine were withdrawn due to the Russian/Ukrainian conflict; these patients were included within the physician decision withdrawal category. The patient disposition, including reasons for withdrawal, is presented in Supplementary Figure 1.

### Benefit:risk assessment

In the majority of study visits, physicians judged that the benefit:risk assessment supported continued mepolizumab treatment. A total of 5 (<1%) patients (*n* = 514) throughout the study were deemed as having a negative benefit:risk ratio by the treating physician, 1 at each of the following study visits: Week 12, 24, 144 and 156 (both the same individual), 168, and 324 (Figure 1(b)). In one case, at Week 48, the physician benefit:risk decision was recorded as ‘unknown’ (missed). There may have been some benefit:risk decisions made between these visits, that would not have been captured by this analysis; however, all decisions are reflected within the overall patient withdrawals from the study (Figure 1(a)).

### Baseline demographics

Overall, 57% of patients were female, 95% were Caucasian, and the mean age (standard deviation, [SD]) was 51.1 (14.9) years. There were 24 (5%) pediatric/adolescent patients 6–17 years of age (Table 1). Patients were included from Europe (296, 58%) and the rest of the world (218, 42%).

**Table 1.** Baseline demographics.

Demographic characteristic	Mepolizumab 100 mg SC (*N* = 502)	Mepolizumab 40 mg SC (*N* = 7)	Mepolizumab 40/100 mg SC (*N* = 5)	Mepolizumab SC (*N* = 514)
Sex, *n*(%)				
Female	290 (58)	2 (29)	1 (20)	293 (57)
Male	212 (42)	5 (71)	4 (80)	221 (43)
Age (years),* mean (SD)	52.1 (13.6)	9.3 (0.8)	8.4 (0.6)	51.1 (14.9)
Age group (years)*, *n*(%)				
6–11	3 (<1)	7 (100)	5 (100)	15 (3)
12–17	9 (2)	0	0	9 (2)
18–64	408 (81)	0	0	408 (79)
≥65	82 (16)	0	0	82 (16)
Ethnicity, *n*(%)				
Hispanic or Latino	43 (9)	1 (14)	0	44 (9)
Not Hispanic or Latino	459 (91)	6 (86)	5 (100)	470 (91)
Race, *n*(%)				
White	481 (96)	3 (43)	4 (80)	488 (95)
Asian	3 (<1)	3 (43)	0	6 (1)
Black or African American	4 (<1)	1 (14)	0	5 (<1)
Other^†^	14 (3)	0	1 (20)	15 (3)

*Age was imputed when full date of birth was not provided. ^†^’Other’ included American Indian, Native Alaskan, Native Hawaiian, Other Pacific Islander or multiple race reported.

SC: subcutaneous; SD: standard deviation.

### Exposure

**Figure 2.**
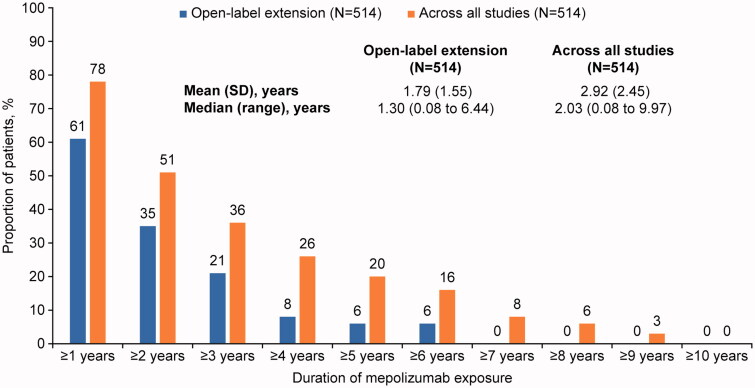
Mepolizumab exposure in the open-label extension and across all mepolizumab clinical studies. SD: standard deviation.

Mean exposure (SD) in this OLE was 1.79 (1.55) years, which equated to a median exposure of 1.30 (range 0.08 to 6.44) years (Figure 2). Including the prior clinical trials and OLEs, mean exposure was 2.92 (2.45) years (median 2.03 [0.08 to 9.97] years). The maximum length of patient exposure in the LAP was 6.44 years and was 9.97 years when the previous clinical- and open-label trials were also considered, inclusive of interruptions in dosing with a given study. Fifteen (3%) patients had a length of exposure ≥9 years. In the patients across all mepolizumab studies, there was a total mepolizumab exposure of 1500.59 patient-years (PYs) at differing dose levels.

### Safety

#### SAEs

Overall, 37/514 (7%) patients experienced on-treatment (within 28 days of dosing) SAEs, 34/502 (7%) in the mepolizumab 100 mg SC group, and 3/7 (43%) in the mepolizumab 40 mg SC pediatric dose group. Only two patients experienced SAEs that were considered treatment-related by the investigator in the mepolizumab 100 mg SC group (single unresolved events of colitis, gastritis, decreased appetite, and colorectal adenoma in one patient while on-treatment and single resolved event of spontaneous abortion post-treatment, e.g. >28 days following the last mepolizumab dose, in another) (Table 2). Three (<1%) patients in the mepolizumab 100 mg SC group experienced SAEs leading to permanent discontinuation of study treatment or withdrawal (breast cancer, asthma, and 1 patient reporting both colitis and gastritis, respectively). Four (<1%) patients experienced SAEs leading to dose interruption/delay. There were no fatal SAEs. The most common on-treatment SAEs were asthma (13 [3%] patients) and X-ray-confirmed pneumonia (3 [<1%] patients) (Table 3).

**Table 2.** Overview of AEs.

No. (%) of patients with SAEs	Mepolizumab 100 mg SC (*N* = 502)	Mepolizumab 40 mg SC (*N* = 7)	Mepolizumab 40/100 mg SC (*N* = 5)	Mepolizumab SC (*N* = 514)
Any on/post-treatment	35 (7)	3 (43)	0	38 (7)
Considered to be treatment-related by investigator	2 (<1)	0	0	2 (<1)
Leading to permanent discontinuation of study treatment	3 (<1)	0	0	3 (<1)
Leading to withdrawal from the study	3 (<1)	0	0	3 (<1)
Leading to dose interruption/delay	4 (<1)	0	0	4 (<1)
Fatal	0	0	0	0
Any on-treatment	34 (7)	3 (43)	0	37 (7)
Any post-treatment	1 (<1)	0	0	1 (<1)
No. (%) of patients with non-serious AEs	Mepolizumab 100 mg SC (*n* = 76)	Mepolizumab 40 mg SC (*n* = 7)	Mepolizumab 40/100 mg SC (*n* = 5)	Mepolizumab SC (*n* = 88)
Any on/post-treatment	43 (57)	6 (86)	3 (60)	52 (59)
Considered to be treatment-related by investigator	5 (7)	1 (14)	0	6 (7)
Leading to permanent discontinuation of study treatment	1 (1)	0	0	1 (1)
Leading to withdrawal from the study	1 (1)	0	0	1 (1)
Leading to dose interruption/delay	4 (5)	0	0	4 (5)
Any on-treatment	43 (57)	6 (86)	3 (60)	52 (59)
Any post-treatment	2 (3)	0	0	2 (2)

AE: adverse event; SAE: serious adverse event; SC: subcutaneous.

**Table 3.** Most frequent on-treatment SAE and non-serious AE preferred terms.

No. (%) of patients with SAE*	Mepolizumab 100 mg SC		Mepolizumab 40 mg SC		Mepolizumab 40/100 mg SC		Mepolizumab SC	
	*N* = 502, *n*(%)	PY = 892.79^†^	*N* = 7, *n*(%)	PY = 11.13^†^	*N* = 5, *n*(%)	PY = 15.85^†^	*N* = 514, *n*(%)	PY = 919.77^†^
Asthma	1(2)	22.4	1(14)	89.8	0	0.0	13(3)	22.8
Pneumonia	2(<1)	2.2	1(14)	89.8	0	0.0	3(<1)	3.3
No. (%) of patients with non-serious AE^‡^	Mepolizumab 100 mg SC		Mepolizumab 40 mg SC		Mepolizumab 40/100 mg SC		Mepolizumab SC	
	*n* = 76, *n*(%)	PY = 107.67^†^	*n* = 7, *n*(%)	PY = 11.13^†^	*n* = 5, *n*(%)	PY = 15.85^†^	*n* = 88, *n*(%)	PY = 134.65^†^
Asthma	23 (30)	334.4	2 (29)	269.5	1 (20)	126.2	26 (30)	304.5
Lower RTI	9 (12)	102.2	0	0.0	1 (20)	63.1	10 (11)	89.1
Headache	8 (11)	278.6	1 (14)	89.8	0	0.0	9 (10)	230.2
Nasopharyngitis	5 (7)	65.0	2 (29)	179.7	0	0.0	7 (8)	66.8
Sinusitis	5 (7)	46.4	1 (14)	89.8	1 (20)	63.1	7 (8)	52.0
Upper RTI	5 (7)	74.3	2 (29)	179.7	0	0.0	7 (8)	74.3
Cough	5 (7)	55.7	0	0.0	0	0.0	5 (6)	44.6
Influenza	1 (1)	9.3	3 (43)	359.3	1 (20)	126.2	5 (6)	52.0
Bronchitis	2 (3)	18.6	0	0.0	2 (40)	189.3	4 (5)	37.1
Pharyngitis	1 (1)	9.3	1 (14)	89.8	2 (40)	315.5	4 (5)	52.0
RTI	4 (5)	55.7	0	0.0	0	0.0	4 (5)	44.6

*All other SAE preferred terms were reported in a single patient. ^†^Numbers represent the number of events per 1000 PYs of exposure.

^‡^All other non-serious AE preferred terms were reported in ≤4% of patients.

AE: adverse event; PY: patient-years; RTI: respiratory tract infection; SAE: serious adverse event; SC: subcutaneous.

#### Non-serious AEs

In the smaller population in whom non-serious AEs were required to be collected, 52/88 (59%) experienced on-treatment, non-serious AEs overall, and rates were higher in the mepolizumab 40 mg SC pediatric dose group (6/7 [86%]) compared with the adult dose (43/76 [57%] or 40/100 mg transition group (3/5 [60%]) (Table 2). Six (7%) non-serious AEs were considered treatment-related by the investigator: 5 (7%; influenza-like illness, injection-site reaction, parosmia, hypersensitivity, nasal congestion, rhinorrhea, and sneezing) in the mepolizumab 100 mg SC group and 1 (14%; headache) with the mepolizumab 40 mg SC pediatric dose. The most frequent non-serious AEs affecting >5% of patients were asthma, lower respiratory tract infection (RTI), headache, nasopharyngitis, sinusitis, upper RTI, cough, and influenza (Table 3).

#### AESIs

Infections, affecting 9/514 (2%) patients, were the most commonly reported serious AESI (Table 4). Other serious AESIs such as neoplasms, malignancies, serious cardiac, vascular and thromboembolic events, and serious ischemic events affected <1% of patients. Infections were also the most commonly reported non-serious AESI. One (1%) patient reported a potential opportunistic infection of herpes zoster. Other non-serious AESIs, including cardiac disorders, were reported in <2% of patients (Table 4).

**Table 4.** On-treatment serious and non-serious AESIs.

No. (%) of patients with serious AESI	Mepolizumab 100 mg SC (*N* = 502)	Mepolizumab 40 mg SC (*N* = 7)	Mepolizumab 40/100 mg SC (*N* = 5)	Mepolizumab SC (*N* = 514)
Any SAE	34 (7)	3 (43)	0	37 (7)
Systemic reactions	0	0	0	0
Allergic reactions	0	0	0	0
Non-allergic reactions	0	0	0	0
Anaphylaxis	0	0	0	0
Local injection-site reactions	0	0	0	0
All infections*	7 (1)	2 (29)	0	9 (2)
Potential opportunistic infections^†^	0	0	0	0
Neoplasms*	3 (<1)	0	0	3 (<1)
Malignancies^‡^	1 (<1)	0	0	1 (<1)
Cardiac disorders*	3 (<1)	0	0	3 (<1)
Serious CVT events^§^	4 (<1)	0	0	4 (<1)
Serious ischemic events^║^	2 (<1)	0	0	2 (<1)
No. (%) of patients with non-serious AESIs	Mepolizumab 100 mg SC (*n* = 76)	Mepolizumab 40 mg SC (*n* = 7)	Mepolizumab 40/100 mg SC (*n* = 5)	Mepolizumab SC (*n* = 88)
Any non-serious AE	43 (57)	6 (86)	3 (60)	52 (59)
Systemic reactions	1 (1)	0	0	1 (1)
Allergic reactions	1 (1)	0	0	1 (1)
Non-allergic reactions	0	0	0	0
Anaphylaxis	0	0	0	0
Local injection-site reactions	1 (1)	0	0	1 (1)
All infections*	32 (42)	5 (71)	3 (60)	40 (45)
Potential opportunistic infections^†^	1 (1)	0	0	1 (1)
Neoplasms*	0	0	0	0
Malignancies^‡^	0	0	0	0
Cardiac disorders*	2 (3)	0	0	2 (2)

*Infections from Infections and infestations SOC, Neoplasms from Neoplasms benign malignant and unspecified (including cysts and polyps) SOC, Cardiac disorders from Cardiac disorders SOC. ^†^Identified from SMQ or events with the preferred term of Herpes Zoster. ^‡^Identified from standard MedDRA queries (SMQs). ^§^Serious CVT events identified from Cardiac Disorders SOC, Vascular Disorders SOC and SMQs. ^║^Subset of serious CVT events identified through SMQs.

AE: adverse event; AESI: adverse event of special interest; CVT: cardiac, vascular and thromboembolic; MedDRA: Medical Dictionary for Regulatory Activities; SC: subcutaneous; SMQ: standard MedDRA query; SOC: system organ class.

#### Safety in the pediatric population

SAEs were reported in 3/15 (20%) of patients 6–11 years of age and 2/9 (22%) patients 12–17 years of age (Table 5). The proportion of pediatric/adolescent patients with SAEs was higher than in the adult age groups, although caution is advised in drawing conclusions based on this as there were only 15 and 9 patients in these pediatric/adolescent age groups, respectively. In the pediatric group, SAEs were reported in the respiratory, thoracic and mediastinal disorders, infections and infestations, and endocrine disorders system organ class (SOC) categories. In the larger population of adult patients, SAEs were reported in a wider range of SOC categories. The only AESIs reported in the pediatric/adolescent population were infections; 3 patients with an infection classified as serious and 12 classified as non-serious (Supplementary Table 2).

**Table 5.** On-treatment SAEs by age group.

No. (%) of patients with SAE	Mepolizumab SC				
	6–11 years (*n* = 15)	12–17 years (*n* = 9)	18–64 years (*n* = 408)	≥65 years (*n* = 82)	Total (*N* = 514)
Any	3 (20)	2 (22)	23 (6)	9 (11)	37 (7)
Respiratory, thoracic. and mediastinal disorders	2 (13)	0	10 (2)	2 (2)	14 (3)
Infections and infestations	2 (13)	1 (11)	2 (<1)	4 (5)	9 (2)
Gastrointestinal disorders	0	0	3 (<1)	1 (1)	4 (<1)
Injury, poisoning and procedural complications	0	0	3 (<1)	1 (1)	4 (<1)
Cardiac disorders	0	0	3 (<1)	0	3 (<1)
General disorders and administration site conditions	0	0	2 (<1)	1 (1)	3 (<1)
Neoplasms benign, malignant and unspecified (incl. cysts and polyps)	0	0	2 (<1)	1 (1)	3 (<1)
Endocrine disorders	0	1 (11)	1 (<1)	0	2 (<1)
Metabolism and nutrition disorders	0	0	0	2 (2)	2 (<1)
Hepatobiliary disorders	0	0	1 (<1)	0	1 (<1)
Nervous system disorders	0	0	0	1 (1)	1 (<1)
Skin and subcutaneous tissue disorders	0	0	1 (<1)	0	1 (<1)

SAE: serious adverse event; SC: subcutaneous.

### COVID-19 infections

No COVID-19 infections (serious or non-serious AEs) were reported. It should be noted that only patients within the non-serious AE population (*n* = 88) were required to report non-serious AEs of COVID-19 infection. Hence, unreported non-serious COVID-19 infections may have occurred within the overall safety population (*n* = 514).

## Discussion

This LAP was a large mepolizumab OLE study of over 500 patients with severe asthma with an eosinophilic phenotype. Patients were treated with mepolizumab for up to 6.5 years, not including prior study exposure, and up to ∼10 years including previous clinical studies. The main aim of this study was to enable patients who had participated in previous mepolizumab clinical trials to continue treatment up to mepolizumab commercialization in the patients’ respective countries, while assessing long-term safety. Asthma was the most common SAE (3% of patients) and infections were the most common serious AESI (2% of patients). However, only two SAEs (colitis, gastritis, decreased appetite and colorectal adenoma in one patient and of spontaneous abortion in another) were considered related to treatment by the investigator. A favorable physician benefit:risk assessment was reported throughout the study period.

The use of biologics in severe asthma is a relatively recent phenomenon, with mepolizumab first receiving US and European approval for use in severe asthma with an eosinophilic phenotype in 2015 [22, 23]. Therefore, having long-term safety data for up to ∼10 years is particularly reassuring for physicians and their patients with asthma, who require chronic therapy. Based on the experience with biologics in other therapy areas such as rheumatology, it can be difficult to collect long-term safety data due to loss of therapeutic response and increased infusion reactions caused by anti-drug antibodies [33], which is likely to result in treatment switching. Therefore, the results of this study, which indicate that mepolizumab has a favorable safety profile with long-term exposure, are particularly important.

These data extend the long-term safety follow-up of mepolizumab that has previously been reported for the COLUMBA study, an OLE of up to 4.5 years for patients from the DREAM double-blind study [34], the COSMOS study, a 52-week OLE of 2 previous double-blind studies, MENSA and SIRIUS [35], and for the COSMEX study, an OLE of up to 172 weeks for patients from COSMOS [36] (Supplementary Table 1). The present long-term access study reports on patients with up to ∼10 years of cumulative exposure to mepolizumab to date, more than 5 years longer than the next longest study. In COLUMBA [34], median exposure to mepolizumab was 3.84 years (range, 4 weeks to 4.54 years); in MENSA, SIRIUS and COSMOS combined [35], median cumulative exposure was 1.47 years (range, 0.08 to 1.83 years) and in MENSA, SIRIUS, COSMOS, and COSMEX combined [36], it was 3.6 years (range, 1.2 to 4.8 years). While the median duration of treatment in the current study is shorter than the median duration of the COLUMBA study, all patients in the COLUMBA study had discontinued by 4.5 years, whereas in this study there is a proportion of patients that continued to receive treatment for significantly longer, up to ∼10 years. A total of 139/514 (27%) and 115/514 (22%) participants reported mepolizumab treatment with a duration exceeding the median and maximum values observed in COLUMBA. When considering only the 49 participants enrolled from the COLUMBA study, all exceeded the maximum mepolizumab treatment duration observed in COLUMBA (4.5 years).

The total mepolizumab exposure in the current study and all preceding studies equates to over 1500 PYs. It should be noted that DREAM, MENSA, SIRIUS, MUSCA, COLUMBA, COSMOS, and COSMEX were all feeder studies for this LAP, hence this study reports longer-term safety data from these same patients. The length of follow-up, nearly 10 years in the current study, is also longer than that of similar extension studies of benralizumab 30 mg (administered monthly or every 2 months; BORA extension and MELTEMI OLE), another asthma biologic targeting the IL-5 pathway; in this analysis, the longest follow-up among adult patients was 5 years (total exposure 1581.3 PYs) [37].

In agreement with the safety observed in earlier mepolizumab long-term OLE studies [34–36], no new safety signals were observed with mepolizumab over the increased follow-up period of up to ∼10 years. As in the previous mepolizumab studies, the most common SAE experienced by patients was asthma, and treatment-related SAEs were rare [34–36]. In the current study, the most common AESI was infections, with serious infections reported in 2% and non-serious infections reported in 45% of patients. Infections were also the most common AESI in the COLUMBA and COSMEX studies, where they were reported in 82% and 83% of patients, respectively [34, 36]. The AESI of serious infections was reported in 5% of patients in COLUMBA [34], 6% of patients in COSMEX [36], and 2% of patients in the current study, suggesting a low risk of serious infections. In previous mepolizumab OLE studies, up to 12% of patients experienced local injection-site reactions [34–36], whereas in the current study, just 1% reported such reactions. A 5-year follow-up study of benralizumab also reported no new safety signals [37]. As with the current study, the most common SAEs with benralizumab were worsening of asthma and infections [37]. It should also be noted that while this was primarily a study to assess the long-term safety of mepolizumab, and therefore lacks any efficacy measure, the need for patients to have a favorable benefit:risk assessment at regular periods throughout the study in order to continue treatment provides reassurance on the durability of efficacy of mepolizumab.

The CV risks associated with mepolizumab appeared low in the current study, and were consistent with a population of this age and body mass index, with <1% experiencing serious cardiac, vascular, thromboembolic, or ischemic events, and 2% experiencing non-serious cardiac disorders. In a randomized, double-blind, placebo-controlled, parallel-group trial of dupilumab 200 mg (*N* = 631), dupilumab 300 mg (*N* = 633), or placebo (*N* = 638), there was a numerical imbalance of SAEs categorized as cardiac disorders, although an expert panel who were blinded to the treatment groups concluded that there was no imbalance [38]. CV risk has also been assessed in patients with severe uncontrolled asthma treated in a randomized, double-blind trial of tezepelumab or placebo, who subsequently moved into an OLE where all patients received tezepelumab. Including both on-treatment events in the placebo followed by tezepelumab group as well as on-study events in both treatment arms, the pooled on-study incidence of cardiac disorder SAEs was 1.30/100 PYs with tezepelumab versus 0.23/100 PYs with placebo, although neither the investigators or a masked independent adjudication committee ascribed causality to tezepelumab for any of these events [39].

This study also gave the opportunity to expand upon mepolizumab safety data for the pediatric population. While just 15 pediatric patients (6–11 years of age) were included in this study, data in this group are consistent with previous observations within this age group [30, 31]. Of the nine adolescent patients (12–17 years of age), rates of serious AESIs and AESIs appeared higher than in adult patients, although patient numbers were low, so caution is advised in drawing conclusions. Additionally, these results do not agree with an earlier study, which reported a similar safety profile for mepolizumab in adult and pediatric patients [32]. While the current study did not assess efficacy, earlier studies in adult, pediatric, and adolescent patients suggested that the efficacy of mepolizumab is similar across age groups [32, 40].

The strengths of the study include the large adult patient population and the length of follow-up, with over 1500 PYs of exposure. Therefore, the results are widely generalizable to an adult patient population with severe asthma. This study also has several limitations. While this is one of the first real-world, long-term safety studies of mepolizumab in a pediatric patient population, the data are limited by the low number of pediatric and adolescent patients. The pediatric data available in this study were restricted by the pool of patients enrolled within prior studies within the mepolizumab clinical development program. Additionally, although there was only a single (non-serious) opportunistic infection over approximately 1500 PYs of mepolizumab exposure, pediatric patients in less developed countries may have differential risk and exposure to parasitic infections than in the current study [41, 42]. Consequently, caution should be exercised when drawing conclusions from the pediatric data. In addition, the design of this study was such that patients who responded to mepolizumab and did not have AEs resulting in discontinuation from earlier trials were more likely to continue treatment, which may have biased the results for positively affected long-term outcomes. In terms of the exposure data, if dosing interruptions occurred during a study, those interruptions may not have been accounted for and exposure may have been overestimated. In contrast, dosing interruptions between studies, such as the enforced 18-month dosing gap between DREAM and COLUMBA, were accounted for. Another potential limitation was that some of the prior mepolizumab dosing was based on non-approved regimens, as in the DREAM and MENSA studies mepolizumab was dosed as 75, 250, or 750 mg intravenously every 4 weeks [24, 26]. Finally, the length of follow-up reflects the speed of approval and commercial availability of mepolizumab in individual countries, so follow-up was shorter in countries with faster approval of mepolizumab, where patients would withdraw from study participation.

In conclusion, this study provides a large, long-term dataset describing the safety of mepolizumab. The safety profile of mepolizumab in severe asthma in this study aligns with previous reports and no new safety signals were observed [34–36]. Mepolizumab treatment was well tolerated, with the majority of patients having a favorable benefit:risk for up to ∼10 years, supporting its long-term use in patients with severe asthma.

## Supplementary Material

Supplemental Material

## Data Availability

The data that support the findings of this study are available from the corresponding author, IP, upon reasonable request.
