# Supplementary material for: Long-term safety of mepolizumab for up to ∼10 years in patients with severe asthma: open-label extension study
Source: Ann Med. 2024 Oct 28;56(1):2417184. doi: 10.1080/07853890.2024.2417184 (PMC11520089; doi:10.1080/07853890.2024.2417184)
Supplement: Supplemental Material [file IANN_A_2417184_SM6801.zip › Suppl_data/Supplementary Material_Final.docx]

**Supplementary Table 1.** Pathway of studies feeding into the current study

| **Pathway of feeder studies into the current study (201956, 2015-22)** | **Study design** | **Mepolizumab dose (every 4 weeks)** | **Study start and end dates** | **Patient numbers by mepolizumab formulation in current study (201956, NCT00244686)** | | | |
| --- | --- | --- | --- | --- | --- | --- | --- |
|  |  |  |  | **100 mg SC (N=502)** | **40 mg SC (N=7)** | **40/100 mg SC (N=5)** | **SC (N=514)** |
| 1. DREAM (NCT01000506) ([23](#_ENREF_23)) 2. COLUMBA (NCT01691859) ([33](#_ENREF_33)) | Phase IIb, randomized, double blind, placebo controlled  Phase III, open label | 75, 250, or 750 mg IV 100 mg SC | 2009–12  2012–17 | 18 | 0 | 0 | 18 |
| 1. DREAM (NCT01000506) ([23](#_ENREF_23)) 2. COLUMBA (NCT01691859) ([33](#_ENREF_33)) 3. COMET (NCT02555371) ([42](#_ENREF_42)) | Phase IIb, randomized, double blind, placebo controlled  Phase III, open label  Phase IIIb, randomized, double blind, placebo controlled | 75, 250, or 750 mg IV  100 mg SC  100 mg SC | 2009–12  2012–17  2016–19 | 31 | 0 | 0 | 31 |
| 1. SIRIUS (NCT01691508) ([26](#_ENREF_26)) 2. COSMOS (NCT01842607) ([34](#_ENREF_34)) 3. COSMEX (NCT02135692) ([35](#_ENREF_35)) | Phase III, randomized, double blind, placebo controlled  Phase IIIb, open label  Phase IIIb, open label | 100 mg SC  100 mg SC  100 mg SC | 2012–13  2013–15  2014–17 | 15 | 0 | 0 | 15 |
| 1. MENSA (NCT01691521) ([25](#_ENREF_25)) 2. COSMOS (NCT01842607) ([34](#_ENREF_34)) 3. COSMEX (NCT02135692) ([35](#_ENREF_35)) | Phase III, randomized, double blind, placebo controlled  Phase IIIb, open label  Phase IIIb, open label | 75 mg IV or 100 mg SC  100 mg SC  100 mg SC | 2012–14  2013–15  2014–17 | 16 | 0 | 0 | 16 |
| 1. MENSA (NCT01691521) ([25](#_ENREF_25)) 2. COSMOS (NCT01842607) ([34](#_ENREF_34)) 3. COSMEX (NCT02135692) ([35](#_ENREF_35)) 4. COMET (NCT02555371) ([42](#_ENREF_42)) | Phase III, randomized, double blind, placebo controlled  Phase IIIb, open label  Phase IIIb, open label  Phase IIIb, randomized, double blind, placebo controlled | 75 mg IV or 100 mg SC  100 mg SC  100 mg SC  100 mg SC | 2012–14  2013–15  2014–17  2016–19 | 11 | 0 | 0 | 11 |
| 1. MUSCA (NCT02281318) ([27](#_ENREF_27)) | Phase IIIb, randomized, double blind, placebo controlled | 100 mg SC | 2014–16 | 384 | 0 | 0 | 384 |
| 1. Pediatric study (NCT02377427) | Phase II, open label (6–11 years of age) | 40 or 100 mg SC | 2015–18 | 8 | 7 | 5 | 20 |
| 1. OSMO (NCT02654145) ([43](#_ENREF_43)) | Phase IV, open label, single arm | 100 mg SC | 2016–17 | 19 | 0 | 0 | 19 |

Data are n. Studies listed in order of enrollment prior to entry into study 201956 with 1= the earliest study in each case. The information after each study name includes study number and the year of study start and study end.

IV, intravenous; SC, subcutaneous.

**Supplementary Table 2.** On-treatment serious and non-serious AESIs by age group

| **No. (%) of patients with serious AESI** | **Mepolizumab SC (N=514)** | | | |
| --- | --- | --- | --- | --- |
|  | **6–11 years (n=15)** | **12–17 years (n=9)** | **18–64 years (n=408)** | **≥65 years (n=82)** |
| Any SAE | 3 (20) | 2 (22) | 23 (6) | 9 (11) |
| Systemic reactions | 0 | 0 | 0 | 0 |
| Allergic reactions | 0 | 0 | 0 | 0 |
| Non-allergic reactions | 0 | 0 | 0 | 0 |
| Anaphylaxis | 0 | 0 | 0 | 0 |
| Local injection-site reactions | 0 | 0 | 0 | 0 |
| All infections* | 2 (13) | 1 (11) | 2 (<1) | 4 (5) |
| Potential opportunistic infections^†^ | 0 | 0 | 0 | 0 |
| Neoplasms* | 0 | 0 | 2 (<1) | 1 (1) |
| Malignancies^‡^ | 0 | 0 | 1 (<1) | 0 |
| Cardiac disorders* | 0 | 0 | 3 (<1) | 0 |
| Serious CVT events§ | 0 | 0 | 3 (<1) | 1 (1) |
| Serious ischemic events^║^ | 0 | 0 | 1 (<1) | 1 (1) |

| **No. (%) of patients with non-serious AESIs** | **Mepolizumab SC (N=514)** | | | |
| --- | --- | --- | --- | --- |
|  | **6–11 years (n=15)** | **12–17 years (n=5)** | **18–64 years (n=53)** | **≥65 years (n=15)** |
| Any non-serious AE | 10 (67) | 4 (80) | 31 (58) | 7 (47) |
| Systemic reactions | 0 | 0 | 1 (2) | 0 |
| Allergic reactions | 0 | 0 | 1 (2) | 0 |
| Non-allergic reactions | 0 | 0 | 0 | 0 |
| Anaphylaxis | 0 | 0 | 0 | 0 |
| Local injection-site reactions | 0 | 0 | 1 (2) | 0 |
| All infections* | 8 (53) | 4 (80) | 23 (43) | 5 (33) |
| Potential opportunistic infections^†^ | 0 | 0 | 1 (2) | 0 |
| Neoplasms* | 0 | 0 | 0 | 0 |
| Malignancies^‡^ | 0 | 0 | 0 | 0 |
| Cardiac disorders* | 0 | 0 | 2 (4) | 0 |

*Infections from Infections and infestations SOC. Neoplasms from Neoplasms benign malignant and unspecified (including cysts and polyps) SOC.
Cardiac disorders from Cardiac disorders SOC. ^†^Identified from SMQ or events with the preferred term of Herpes Zoster. ^‡^Identified from SMQs.
^§^Serious CVT events identified from Cardiac Disorders SOC, Vascular Disorders SOC and SMQs. ^║^Subset of Serious CVT events identified through SMQs.

AE, adverse event; AESI, adverse event of special interest; CVT, cardiac, vascular and thromboembolic; MedDRA, Medical Dictionary for Regulatory Activities; SC, subcutaneous; SMQ, standard MedDRA query; SOC, system organ class.

**Supplementary Table 3.** List of Independent Ethics Committees and Institutional Review Boards

| **Country** | **Investigator no./Center no.** | **Independent Ethics Committees/Institutional Review Boards** |
| --- | --- | --- |
| Argentina | 126818/232449 | Comite de etica en Investigacion CEMER |
|  | 186807/232450 | Comité Independiente de ética para ensayos en Farm Clinic |
|  | 197304/232452 | Comité de Ética en Investigación CER Salud de la Fundación Respirar Salud |
|  | 021062/219463 | Escuela Latinoamericana de Bioética, Comité de Bioética |
|  | 066860/219464 | Comite de Etica Dr. Claude Bernard |
|  | 065927/219459 | Comite de Etica en Farmacologia Clinica de la Fundacion CIDEA |
|  | 217569/232453 | Comité de ética en investigación CECIC |
|  | 229648/219460; 025953/219458 | Investigaciones en Alergia y Enfermedades Respiratorias |
|  | 144351/219462 | Ave Pulmo |
| Belgium | 244317/218032; 087856/218033; 030058/218034; 003130/218035; 248460/218036 | UZ Gent, Ethisch Comité |
| Bulgaria | 009906/220347; 027904/220346 | Ethics Committee for Multicenter Clinical Trials |
| Canada | 003955/219570 | Institutional Review Board Services |
|  | 006412/218108 | Conjoint Health Research Ethics Board (CHREB) |
|  | 006610/218109 | University of British Columbia, Clinical Research Ethics Board |
|  | 007619/218110; 007707/218111 | Comité d’éthique de la recherche de l’HSCM |
|  | 220890/219140 | IRB Services |
| Czechia | 267717/218418 | FN a LF UP Olomouc, Eticka komise |
|  | 188143/218422; 000346/218419 | FN Hradec Kralove, Eticka komise |
|  | 267712/218417; 000590/232021; 344676/232020; 106575/232022 | Fakultní nemocnice Hradec Kralove |
|  | 054159/218423 | Slezska nemocnice v Opave |
|  | 347564/218420 | FN Plzen |
| Estonia | 222889/219736; 001022/219735; 000976/219737 | National Institute for Health Development |
| France | 346063/219328; 170210/219329; 080238/219330; 001215/219331; 178531/233715; 117455/219332; 246266/219334; 181688/219333; 192135/231769; 003020/233717; 136883/219327; 348739/219335; 218224/219326; 001324/233716 | CPP Ouest IV |
| Germany | 317630/218999; 005964/219000; 197933/219003; 116973/219004; 189487/219005; 003841/219006; 003796/219007; 198174/219008 | Ärztekammer Schleswig-Holstein, Ethikkommissionen |
| Greece | 004048/218729; 346725/218731; 136509/218744; 221411/218730; 003853/218746; 002534/218745 | National Ethics Committee |
| Italy | 082245/219996 | Comitato Etico dell'Azienda Ospedali Riuniti di Foggia |
|  | 185411/219992 | Comitato Etico per Parma |
|  | 344286/219993 | Comitato Etico Area 2 – AOU Consorziale Pol. Bari |
|  | 231245/219995 | Comitato Etico Reg. Toscano Area Vasta Centro |
| Japan | 266909/231742; 207959/231741 | National Hospital Organization, Central Review Board |
|  | 118136/231430 | Fukuyama City Hospital |
| Netherlands | 266850/218090; 266855/218091; 266852/218092; 221227/218093; 332138/218094 | IRB/EC Catharina Ziekenhuis |
| Norway | 345396/219477 | Regionale komiteer for medisinsk og helsefaglig forskningsetikk REK Vest |
| Peru | 060141/219266; 189999/219268; 021792/219265 | Comite Institucional de Bioteica (CIS) Via Libre |
|  | 075686/219269 | CIEI Institucional del Hosp Nacional Cayetano Heredia |
| Poland | 234539/234458; 204592/231876; 148502/234457; 194724/231874; 001325/231873; 000949/231875 | Komisja Bioetyczna przy Dolnoslaskiej Izbie Lekarskiej we Wroclawiu |
| Russian Federation | 224775/218740 | City Clinical Hospital #40 |
|  | 029749/218738 | Ethics Committee of State Educational Institution of Additional Professional Education |
|  | 119210/218736; 263203/218735; 268095/218737 | Ethics Committee of the LLC PharmacoNadzor |
|  | 187431/233036 | Saint-Petersburgh Medical Academy of Postgraduate Education |
|  | 235420/218734 | 155, Turgeneva street Novosibirsk |
|  | 245979/218739 | Voronezh Regional Clinical Hospital |
|  | 001154/218733 | Ural state medical academy |
|  | 001117/218741 | Independent Ethics Committee PharmNadzor |
| Slovakia | 336561/219221; 055407/219222; 224372/219223 | EK pri VUC Kosice |
| Spain | 351599/218752; 011666/218749; 317519/218751; 354545/218754; 354546/218755; 011352/218753 | None |
| Ukraine | 189617/220222 | Kharkiv City Hospital #13 |
|  | 191620/220212 | City Clinical Hospital #6 |
|  | 076201/220221; 001071/220220; 088353/220209 | Institute of Phthisiatry and Pulmonology, Local Ethic Committee |
|  | 227347/220210 | Communal Institution "Road Clinical Hospital #2" |
|  | 214834/238308 | City Clinical Hospital #1 |
|  | 001087/220211 | Vinnytsia City Clinical Hospital #1 |
|  | 191366/220218 | Municipal Institution "Odesa Regional Clinical Hospital" |
|  | 192836/220219 | Regional clinical hospital n.a. M.I. Pyrohov |
| United Kingdom | 347539/219910; 353106/233286; 267079/219911; 023310/219913; 228193/220074; 349746/232955 | Health Research Authority |
| United States | 129354/219278 | Western-Copernicus Group Institutional Review Board |
|  | 013791/219279; 007907/219282 | Advarra Institutional Review Board |

**Supplementary Figure 1.** Patient Disposition in the Long-Term Access Programme


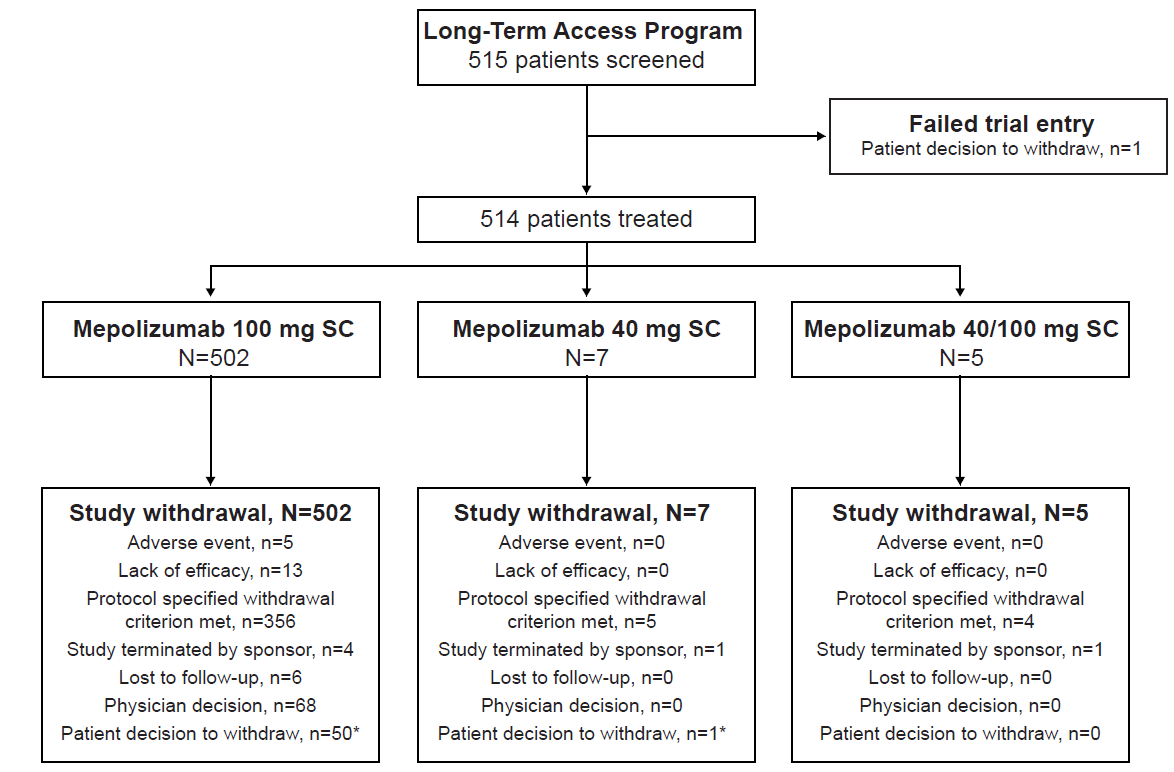


*Reasons for study withdrawal due to “patient decision” were personal/family reasons (n=19), patient relocation (n=11), patients didn’t want to continue (n=5), visits not compatible with work (n=5), patient unwillingness to change site (n=2), transportation issues/travelling problems (n=2), concerned of potential for anaphylaxis (following update to informed consent form; n=2), patient fears COVID-19 pandemic reasons (fear of catching COVID; n=2), or no further explanation/reason provided (n=3).
