# Supplementary figures and images for: Long-term safety of mepolizumab for up to ∼10 years in patients with severe asthma: open-label extension study
Source: Ann Med. 2024 Oct 28;56(1):2417184. doi: 10.1080/07853890.2024.2417184 (PMC11520089; doi:10.1080/07853890.2024.2417184)

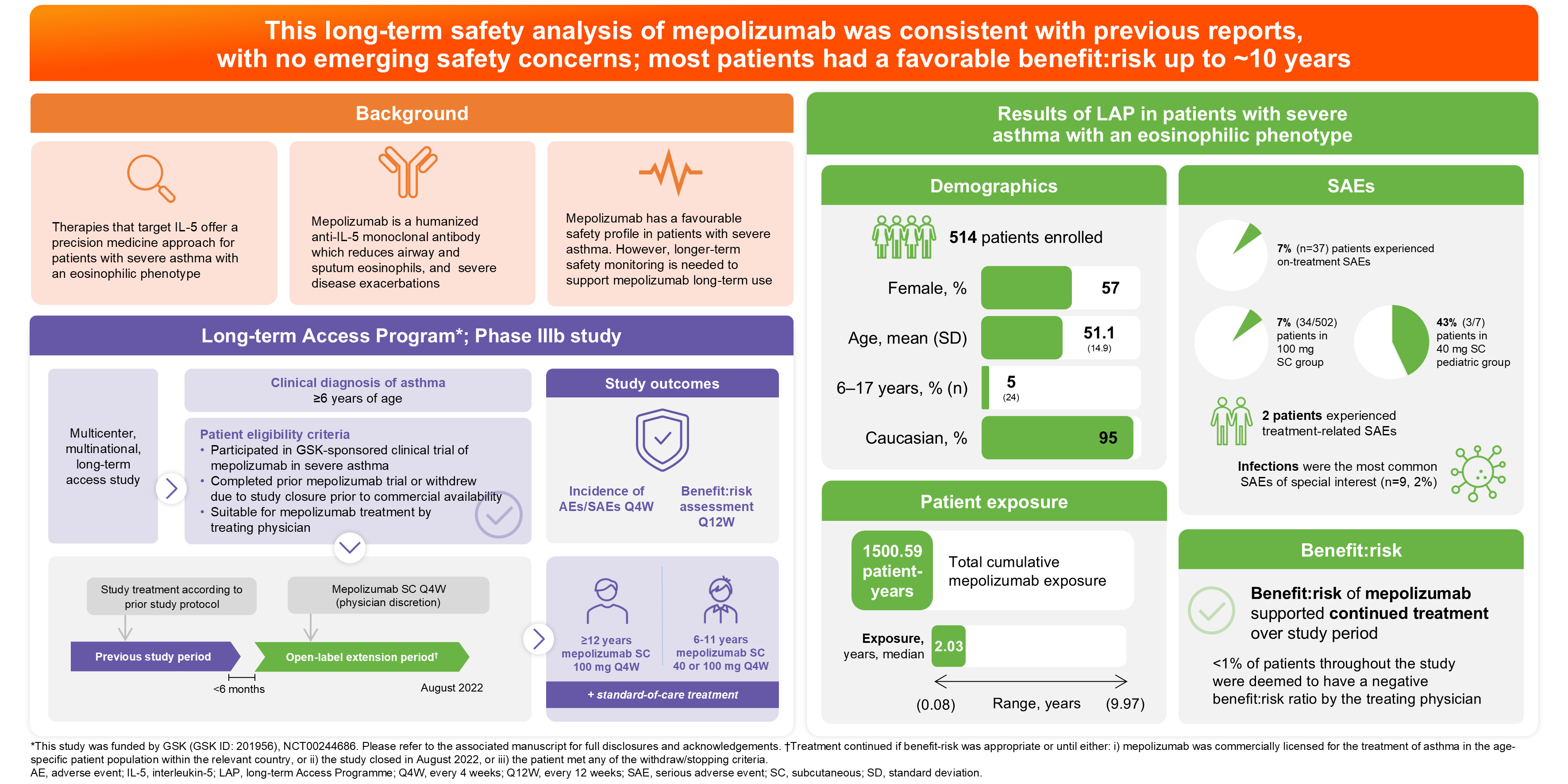

Supplement: Supplemental Material [file IANN_A_2417184_SM6801.zip › Suppl_data/201956_long_term_access_programme_safety_10yr_GA_2Oct.tif]
